# Selection and Validation of Reference Genes for miRNA Expression Studies during Porcine Pregnancy

**DOI:** 10.1371/journal.pone.0028940

**Published:** 2011-12-12

**Authors:** Jocelyn M. Wessels, Andrew K. Edwards, Candace Zettler, Chandrakant Tayade

**Affiliations:** 1 Department of Biomedical Sciences, Ontario Veterinary College, University of Guelph, Guelph, Canada; 2 Department of Biomedical and Molecular Sciences, Queen's University, Kingston, Canada; Baylor College of Medicine, United States of America

## Abstract

MicroRNAs comprise a family of small non-coding RNAs that modulate several developmental and physiological processes including pregnancy. Their ubiquitous presence is confirmed in mammals, worms, flies and plants. Although rapid advances have been made in microRNA research, information on stable reference genes for validation of microRNA expression is still lacking. Real time PCR is a widely used tool to quantify gene transcripts. An appropriate reference gene must be chosen to minimize experimental error in this system. A small difference in miRNA levels between experimental samples can be biologically meaningful as these entities can affect multiple targets in a pathway. This study examined the suitability of six commercially available reference genes (*RNU1A*, *RNU5A*, *RNU6B*, *SNORD25*, *SCARNA17*, and *SNORA73A*) in maternal-fetal tissues from healthy and spontaneously arresting/dying conceptuses from sows were separately analyzed at gestation day 20. Comparisons were also made with non-pregnant endometrial tissues from sows. Spontaneous fetal loss is a prime concern to the commercial pork industry. Our laboratory has previously identified deficits in vasculature development at maternal-fetal interface as one of the major participating causes of fetal loss. Using this well-established model, we have extended our studies to identify suitable microRNA reference genes. A methodical approach to assessing suitability was adopted using standard curve and melting curve analysis, PCR product sequencing, real time PCR expression in a panel of gestational tissues, and geNorm and NormFinder analysis. Our quantitative real time PCR analysis confirmed expression of all 6 reference genes in maternal and fetal tissues. All genes were uniformly expressed in tissues from healthy and spontaneously arresting conceptus attachment sites. Comparisons between tissue types (maternal/fetal/non-pregnant) revealed significant differences for *RNU5A*, *RNU6B*, *SCARNA17*, and *SNORA73A* expression. Based on our methodical assessment of all 6 reference genes, results suggest that *RNU1A* is the most stable reference gene for porcine pregnancy studies.

## Introduction

MicroRNAs (miRNAs) are a recently discovered class of bio-regulatory, short, non-coding molecules that bind to target messenger RNAs (mRNAs) and repress their translation. This is achieved by physically inhibiting translation of the mRNA or through its degradation [1], [2]. MiRNAs participate in various biological processes [3], [ Reviewed in: 4]–[7] and miRNA profiles may be more accurate predictors of disease classification than mRNA profiles [8].

Real time PCR is a sensitive method of measuring gene transcript levels in biological systems. It has recently become a popular method of measuring miRNA expression [9]–[18]. Relative quantification is the preferred method of quantification as absolute quantification has the potential to contain multiple measurement errors. To minimize experimental errors which may occur at any step of the RNA to cDNA to PCR transition or between PCR runs, relative quantification employs a reference gene to normalize the measurement of transcript levels in experimental samples. As miRNA gene expression profiling is an emerging methodology, few reports have been published which identify suitable reference genes for real time PCR studies.

Reference, control, or housekeeping genes as they are also known are genes which express transcripts at uniform levels. The ideal reference gene is constitutively expressed at consistent levels in all samples, tissue types (including physiological and pathological specimens), and altered experimental conditions. Relative quantification creates a ratio of the number of transcripts of a gene of interest with the number of transcripts of the unchanging reference gene within the same sample. This allows samples from different individuals to be more accurately compared because sample variation is standardized. Thus, samples of different qualities, of differing amounts of cDNA, or from different PCR runs can be compared. Unfortunately the expression of many reference genes does change in different tissue types and under different experimental conditions [10], [19]–[21]. As the difference in miRNA levels between experimental samples can be very small, yet still biologically meaningful [10], [22], choosing an unstable reference gene has the potential to mask significant differences [10], [22]. The selection and validation of an appropriate reference gene for every experiment is of paramount importance.

During early gestation in the pig a large number of conceptuses spontaneously arrest [23]–[25]. This presents a challenge when selecting a miRNA reference gene as there are four physiologically and pathologically distinct types of tissues (maternal endometrium and fetal trophoblast associated with healthy conceptus, maternal endometrium and fetal trophoblast associated with arresting or dying conceptuses) present in the same pregnant uterus. This study aims to determine the suitability of six potential reference genes for miRNA quantification by real time PCR during early gestation in the pig. These six genes were selected because human miRNA primers which had been demonstrated effective in other species (mouse, rat, and dog) were commercially available. This is the first report outlining a methodological assessment of several snRNA and snoRNA reference genes for miRNA expression studies in the pig. As such, the methods and results provide useful information and insight to any researcher intent on quantifying miRNAs in other species.

## Results

### Strategy for the Assessment of Reference Genes

A list of requirements was generated to help select the best reference gene (listed in Table 1) for measuring miRNA expression during early gestation in the pig. The most appropriate gene(s) should have: 1) a PCR efficiency close to 2 (within 80–100%), 2) a standard curve where 10-fold dilutions have crossing points (C_p_s) approximately 2.5 cycles apart, 3) a narrow range of C_p_/no significant differences in C_p_s across the tissues of interest, 4) a single melting peak and melting temperature of approximately 74–77°C, 5) its sequence confirmed by sequencing the PCR product, 6) its product size confirmed by gel electrophoresis, 7) an M value of ≤1.5 as determined by geNorm software [26], 8) low inter- and intra-group variation and stability value as determined by NormFinder software [27], and 9) a standard error of mean (SEM).

**Table 1:** Candidate Reference Genes.

Short Form	Full Name	Estimated Size (bp) [33]
RNU1A	U1 small nuclear RNA	125
RNU5A	U5A small nuclear 1 RNA	130
RNU6B	U6 small nuclear 2 RNA	100
SNORD25	Small nucleolar RNA, C/D box 25	130
SCARNA17	Small Cajal body-specific RNA 17	125
SNORA73A	Small nucleolar RNA, H/ACA box 73A	150

### Real Time PCR, Cloning and Sequencing of Candidate Reference Genes

**Figure 1:**
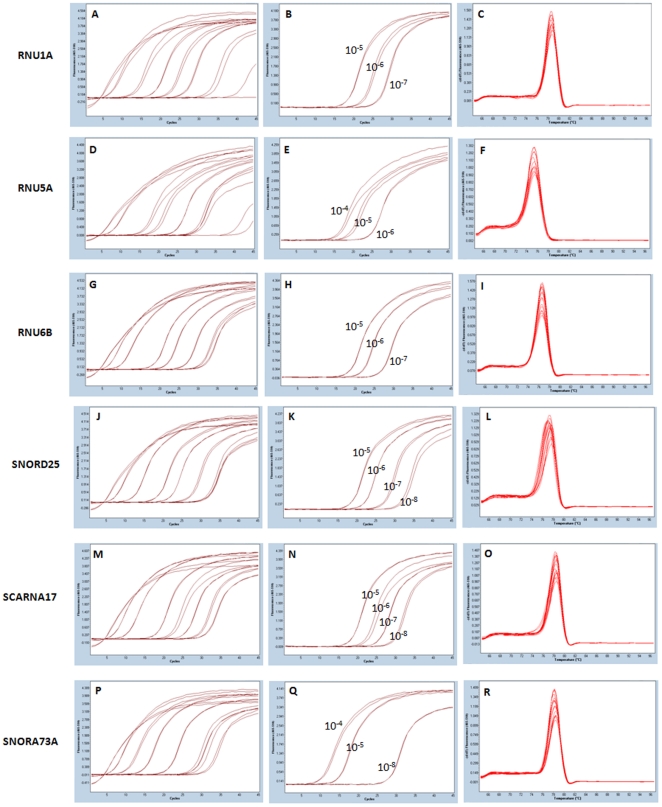
Standard Curves and Melting Peaks of Candidate Genes. Standard curves were generated with a 10-fold dilution for each reference gene (A, D, G, J, M, P). Several dilutions were removed to optimize PCR efficiency (B, E, H, K, N, Q). Melting curve analysis revealed six single peaks, and different temperatures (C, F, I, L, O, R). Numbers on the graph indicate 10-fold dilutions remaining in the optimized standard curve.

Standard curves (10-fold dilution) were generated and optimized for each reference gene (Figure 1A, 1B, 1D, 1E, 1G, 1H, 1J, 1K, 1M, 1N, 1P, 1Q) by plate-based real time PCR. All candidate genes had adjusted PCR efficiencies within the 80–100% range, with *SCARNA17* having the greatest efficiency (Table 2). Out of the six candidate genes, the standard curves for *RNU1A* and *SNORD25* were closest to having C_p_s approximately 2.5 cycles apart (Figure 1A, 1J). Melting curves were generated and specific melting temperatures for each gene were calculated (Figure 1C, 1F, 1I, 1L, 1O, 1R), (Table 2). *RNU1A*, *SCARNA17*, and *SNORA73A* had melting temperatures slightly higher than the kit manufacturer's 74–77°C range. No double melting peaks were observed, indicating a pure PCR product (Figure 1C, 1F, 1I, 1L, 1O, 1R). Of the six candidate genes, five (*RNU1A*, *RNU5A*, *RNU6B*, *SNORD25*, and *SCARNA17*) were successfully cloned, sequenced, and identified as the correct gene using NCBI BLAST analysis. *SNORA73A* could not be cloned, even after three attempts. All six PCR products were run on an agarose gel, and product sizes were within the expected 100–150 bp range [data not shown].

**Table 2:** Summary of Reference Gene Assessment.

Reference Gene	Raw PCR Efficiency	Standard Curve Dilutions Remaining	Adjusted PCR Efficiency	Melting Temperature (°C)	C_p_ Range	Confirmed by Sequencing	Product Size (bp)	Sig. Diff.	M Value	Stability Value
RNU1A	1.647	10^−5^, 10^−6^, 10^−7^	1.762 = 88%	78.49	5.02	Yes	∼150	No	0.320	0.143
RNU5A	1.632	10^−4^,10^−5^, 10^−6^	1.716 = 86%	75.89	5.33	Yes	∼125	Yes	0.521	0.328
RNU6B	1.625	10^−5^, 10^−6^, 10^−7^	1.757 = 88%	76.46	3.82	Yes	∼100	Yes	0.418	0.345
SNORD25	1.634	10^−5^, 10^−6^, 10^−7^, 10^−8^	1.701 = 85%	77.27	4.38	Yes	∼125	No	0.345	0.186
SCARNA17	1.678	10^−5^, 10^−6^, 10^−7^, 10^−8^	1.855 = 93%	78.20	5.23	Yes	∼150	Yes	0.663	0.383
SNORA73A	0.640	10^−4^,10^−5^, 10^−8^	1.685 = 84%	78.25	5.13	No	∼150	Yes	0.331	0.153

### Measurement of Reference Gene Expression in a Panel of Experimental Samples

**Figure 2:**
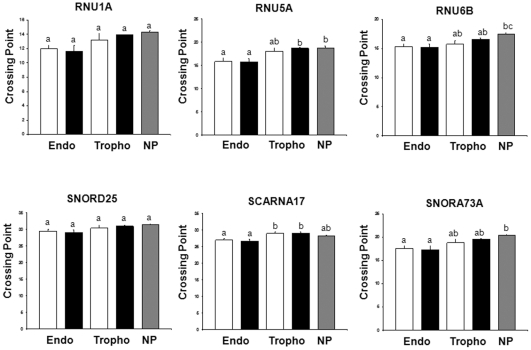
Real Time PCR Crossing Point Values in Pig Reproductive Tissues. Expression levels of all six candidate genes, shown as the mean crossing point (C_p_) plus SEM for each tissue type. White bars: tissue collected from healthy conceptus attachment sites, black bars: tissue collected from arresting conceptus attachment sites, grey bars: non-pregnant endometrium. Means were compared by ANOVA and significant differences (p<0.05) are indicated by different letters above the bars of the graph. If bars have a letter in common, no significant difference exists. For all white and black bars, n = 3. For grey bars, n = 4. Endo: endometrium; NP: non-pregnant endometrium; Tropho: Trophoblast.

Plate-based real time PCR was used to measure the expression of all six reference genes in a panel of sixteen samples which included five tissue types (healthy maternal endometrium (HE), healthy fetal trophoblast (HT), arresting maternal endometrium (AE), arresting fetal trophoblast (AT), and non-pregnant endometrium (NP)) (Figure 2). C_p_ values were measured in duplicate by Roche LightCycler software. The six reference genes displayed a wide range of C_p_ values from 11.25 (*RNU1A*) to 32.36 (*SNORD25*). Both *SNORD25* and *SCARNA17* had low levels of expression, whereas *RNU1A*, *RNU5A*, *RNU6B* and *SNORA73A* had high levels of expression (Figure 2). The C_p_ range for each gene was calculated by subtracting the lowest C_p_ value from the highest C_p_ value. *RNU6B* had the narrowest range (Table 2).

Mean amplification C_p_s and SEM were calculated for each tissue type, and data was compared by one-way ANOVA. The overall variation between samples of the same tissue type was low, as was the variability between tissue types. Significant differences (p<0.05) existed between tissue types for *RNU5A*, *RNU6B*, *SCARNA17*, and *SNORA73A* (Figure 2).

### Stability of Reference Genes in Reproductive Tissues

The expression stability of all six candidate genes was measured using two different algorithms which measure variation: geNorm [26] and NormFinder [27]. GeNorm is a mathematical algorithm used to select the most stable reference gene from a panel of genes, where genes with an M value ≤1.5 are stably expressed genes. All six candidate genes had M values less than 1.5 (Table 2). *RNU1A* had the lowest M value, followed by *SNORA73A*, *SNORD25*, *RNU6B*, *RNU5A*, and *SCARNA17*. GeNorm also calculated a V value which recommended the use of two reference genes (*RNU1A* and *SNORA73A*) for the optimal normalization factor [data not shown].

**Figure 3:**
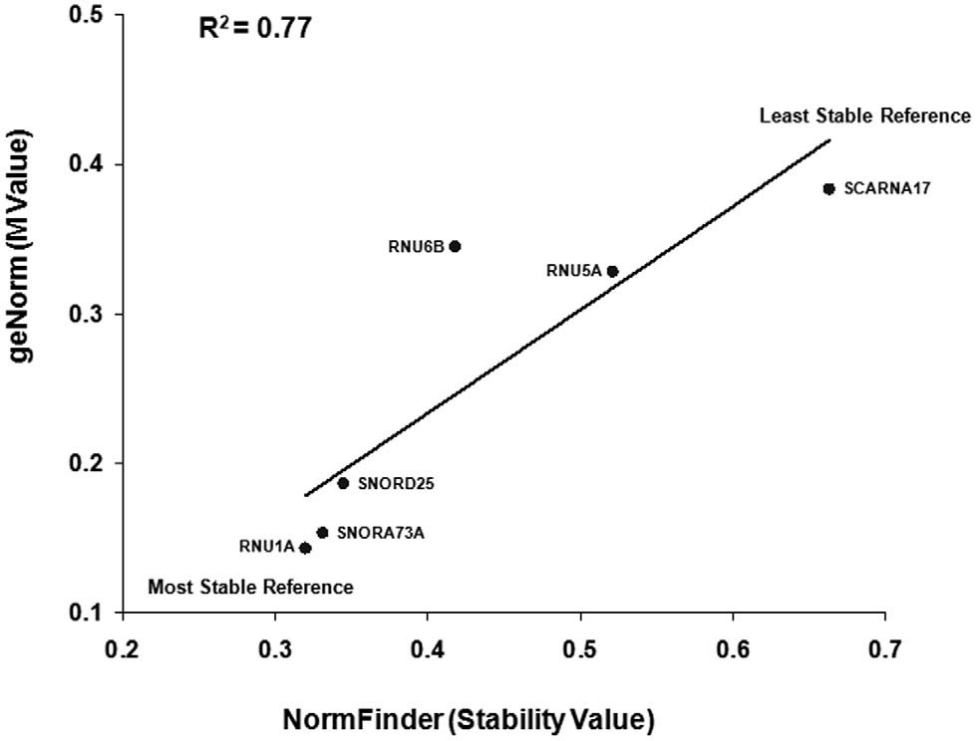
Correlation of M Value and Stability Value. The correlation between the M value calculated by geNorm and Stability Value calculated by NormFinder was evaluated using the coefficient of determination (R^2^).

NormFinder is another algorithm used to select the most stable reference gene from a panel of genes, where genes with a low stability value are stably expressed genes. All six genes had stability values less than 0.4 (Table 2). Remarkably, four out of six genes followed the same rank order of stability as calculated by geNorm. The only exception was that the order of *RNU6B* and *RNU5A* stability was reversed. NormFinder also recommended the use of two reference genes (*RNU1A* and *SNORD25*) [data not shown]. However, the stability value of *RNU1A* combined with *SNORD25* was 0.001 lower than that of *RNU1A* alone. A robust correlation between the geNorm M value and the NormFinder Stability Value was found by evaluating by the coefficient of determination (R^2^) (Figure 3).

### Effect of Reference Gene Selection on Target Gene Expression

**Figure 4:**
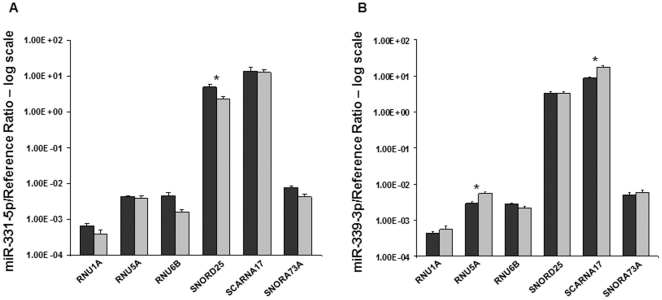
Importance of Reference Gene Stability on the Relative Quantification of Target Genes. Relative quantification of *miR-331-5p* (A) and *miR-339-3p* (B) with each reference gene in healthy endometrium and trophoblast. Differential expression of *miR-331-5p* and *miR-339-3p* is observed, even though microarray data [data not shown] indicated consistent expression between the tissues. Healthy endometrium (HE) was compared to healthy trophoblast (HT) independently for each reference gene by t-test. Data are shown as the mean + SEM, on a logarithmic scale. Dark grey bars: HE (n = 3), light grey bars: HT (n = 3). Significant differences (p<0.05) between HE and HT are demonstrated by asterisk (*) above the bars for the reference gene where the significant difference occurred.

The effect of reference gene selection on target gene expression was measured using *miR-331-5p* and *miR-339-3p* as target genes. A microarray was performed [data not shown] using the same experimental samples, and determined the expression of both genes to be equal in HE and HT. Real time PCR was used to assess the effect a reference gene could have on target quantification. Each target was quantified to each reference gene (Figure 4). Significant differences were observed between target gene expression in HE compared with HT when using *SNORD25* (Figure 4A), *RNU5A*, or *SCARNA17* (Figure 4B) as reference genes. Selection of an inappropriate reference gene had the ability to artificially influence the relative quantity of *miR-331-5p* and *miR-339-3p*.

### Effect of Two Reference Genes on Target Gene Expression

**Figure 5:**
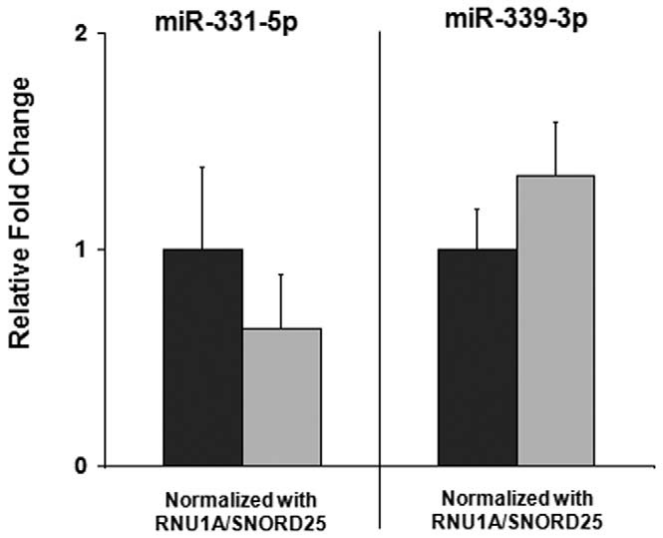
Normalizing Target Genes to the Two Most Stable Reference Genes: *RNU1A/SNORD25*. Fold change for *miR-331-5p* and *miR-339-3p* between healthy endometrium (HE) and trophoblast (HT) were calculated using the geometric mean of *RNU1A/SNORD25* and the ddCt method. HE was compared to HT for each target gene independently by t-test. When the most stable pair of references genes predicted by geNorm and NormFinder algorithms was used as a normalizer, no significant differences in miRNA expression were observed between HE and HT. HE samples were arbitrarily set to a value of 1 + SEM (as a percentage of the variation among biological replicates), HT samples show the fold change above or below HE + SEM. Dark grey bars: healthy endometrium (n = 3), light grey bars: healthy trophoblast (n = 3).

The fold change for *miR-331-5p* and *miR-339-3p* between HE and HT were calculated using the geometric mean of *RNU1A/SNORD25* and the ddCt method (Figure 5). When the most stable pair of references genes as predicted by geNorm and NormFinder was used for quantification, no significant differences were observed.

## Discussion

To allow for the most accurate comparison of miRNA transcripts, it is imperative to select a reference gene with the least amount of variation between samples, and tissues. The optimal gene(s) should be constitutively expressed at similar levels in all tissues, and not influenced by internal or external factors. This is particularly important for miRNA expression profiling because they may be more accurate predictors of disease than mRNA profiles [8]. Even small differences in miRNA expression may be biologically significant because miRNAs regulate multiple targets in a pathway, amplifying their effects [4]. Measuring miRNAs against an unsuitable/unstable reference gene can cause drastic errors of measurement [10], [11]. Peltier and Latham (2008) demonstrated that the use of inappropriate reference genes produced quantitative (magnitude of fold change) and qualitative (direction of fold change) errors, and even supported with statistical significance an incorrect conclusion [10]. This reinforces the need for experiment and tissue-specific validation of reference genes for miRNA expression studies.

While many reference genes for mRNA expression studies have been identified [19]–[21], even for use in the pig [21], [28], no well-established reference genes for miRNA quantification have been identified in this species. Human miRNA studies employ various reference genes including two other small nuclear RNAs RNU48 [12] and RNU44 [18]. However, only a few studies which assess and validate the use of miRNA reference genes in human disease: lung cancer [10], breast cancer [12], and colorectal cancer [11] have been published. Many others have been published prior to adequate reference validation. It is likely that many miRNA reference genes other than those tested in this report exist in the pig. Any reference gene proven effective in another species qualifies for validation as a miRNA reference gene in the pig.

Of the six reference genes tested in this report, two have been employed in other studies. *RNU1A* has been used as a miRNA reference gene in recently published prostate cancer research [15], [16], and *RNU6B* in colorectal cancer [17], tumour pathology [18], erythropoiesis [29], and cell proliferation and growth [30]. Yet, several studies have found *RNU6B* to be an inappropriate/unstable reference gene due to its variability [10], [31], [32]. Our data also suggests that *RNU6B* is an inferior choice of reference gene for miRNA expression profiling during early gestation in the pig because of its altered expression across tissues. Consistent, reliable, and validated miRNA reference genes for expression studies in mammals are absolutely required. This will allow the field of miRNA research to rapidly advance.

This is the first report detailing the validation of suitable reference genes for the normalization of miRNA real time PCR data in pigs. It is also the first to demonstrate the expression of all six reference genes and two target genes (*miR-331-5p*, *miR-339-3p*) in pigs. Here, we compared six different commercially available miRNA reference genes in a panel of five pig reproductive tissues. Selecting an appropriate reference gene for quantification of miRNAs in reproductive tissues is a challenge as there are four physiologically and pathologically distinct types of tissues (maternal endometrium and fetal trophoblast associated with healthy conceptus, maternal endometrium and fetal trophoblast associated with arresting or dying conceptuses) present in the same pregnant uterus. Non-pregnant endometrium was added as the fifth tissue as many comparisons are likely to be made between non-pregnant and pregnant endometrium to demonstrate the molecular fingerprint of pregnancy.

In order to determine the suitability of six human miRNA reference genes in early reproductive tissues in the pig, a strategy for their assessment was developed to systematically eliminate the more unstable and inappropriate genes. The first criterion was that the PCR efficiency was close to 2 (within 80–100%). All six genes tested had efficiencies within this range. The second criterion was that a standard curve where 10-fold dilutions with C_p_s approximately 2.5 cycles apart could be generated. Standard curves for all six genes were generated, and none of the genes tested had standard curves which indicated a problem with amplification of the gene transcript. The third criterion was a narrow range of C_p_/no significant differences in C_p_s across the tissues of interest. Here, several genes were deemed inappropriate for use as a miRNA reference gene in early gestational tissues of the pig. While all genes had a fairly narrow range of C_p_s, several of the putative reference genes had significant differences across the tissues of interest. *RNU5A*, *RNU6B*, *SCARNA17*, and *SNORA73A* are thus not appropriate miRNA reference genes in early pig gestational tissues. The fourth criterion was a single melting peak and melting temperature of approximately 74–77°C. Again, all six genes met this criterion. The fifth criterion was to have the gene sequence confirmed by sequencing the PCR product, to ensure the correct gene transcript was being isolated. The sixth criterion was an M value of ≤1.5 as determined by geNorm software [26]. Again, all six genes met this criterion. The seventh criterion was a low inter- and intra-group variation and stability value as determined by NormFinder software [27]. All six genes met this criterion. As all six genes appeared to isolate a single, specific PCR product, any of them could be used as a miRNA reference gene in the pig, provided they are stably expressed across the tissues of interest.

The most stably expressed reference genes in pig reproductive tissues from both healthy and arresting attachment sites were *RNU1A*, *SNORA73A*, and *SNORD25*, according to geNorm, NormFinder, and regression analysis. Both geNorm and NormFinder algorithms recommended the use of *RNU1A/SNORA73A*, and *RNU1A/SNORD25* respectively, as the most stable pair of reference genes to normalize expression data. However, out of these three genes, both *SNORA73A* and *SNORD25* were shown to be inappropriate reference genes during early porcine gestation. As a consequence of not specifically measuring the inter-tissue variation geNorm inappropriately selected *SNORA73A* as part of the most stable pair of reference genes, even though statistically significant differences between the tissue types were demonstrated by ANOVA and its specific PCR product could not be confirmed. NormFinder selected a combination of *RNU1A/SNORD25* to represent the most stable pair of reference genes for miRNA expression studies by real time PCR during early gestation in the pig. However, when *SNORD25* was used as a reference gene to measure *miR-331-5p* expression between healthy endometrium and trophoblast, an artificial significance not seen in microarray data from the same tissue set, was observed. Indeed, the selection of an inappropriate reference gene had the ability to artificially influence the relative quantity of both *miR-331-5p* and *miR-339-3p*. The observed difference was ablated when the geometric mean of *RNU1A/SNORD25* were used as the normalizing factor. However, when comparing the sensitivity of the ratio of target to reference produced by the LightCycler software, to the fold change manually calculated, the former appeared to have a greater sensitivity to small differences. This is important because the difference in miRNA levels between experimental samples can be very small, yet still biologically meaningful [10], [22].

Although several studies suggest the use of one to three validated reference genes for each individual experiment [26], [27], others demonstrate that one gene may be sufficient for certain experimental situations [10]. As two of the three genes predicted by geNorm and NormFinder were demonstrated in this report to be unstably expressed across porcine reproductive tissues (*SNORA73A*, and *SNORD25*), and the stability value of *RNU1A/SNORD25* was only marginally lower than that of *RNU1A* alone, it appears appropriate to use *RNU1A* as a reference gene for miRNA expression studies during early pregnancy in the pig.

The results of this study indicate the cross-reactivity of all six human miRNA reference primers in pig reproductive tissues. Each of these genes has the potential to be used as a porcine miRNA reference gene provided it is validated and stably expressed in the experimental tissues of choice. *SNORA73A* PCR product remains to be confirmed by sequencing, but the primer is likely isolating a specific product based on melting curve analysis, and gel electrophoresis. The results of this study also demonstrate that reference gene expression can vary between tissue types. Therefore, the selection of a validated and appropriate reference gene(s) for each experiment is the optimal strategy for minimizing errors of measurement in miRNA expression studies. Four of the six reference genes examined were not appropriate for miRNA studies during early gestation in the pig. If two reference genes are absolutely required, *RNU1A/SNORD25* are recommended. However, if one reference gene is required, *RNU1A* is the most stably expressed reference gene for the real time PCR quantification of miRNAs during early porcine gestation, as determined by C_p_/PCR analysis, geNorm, NormFinder, and testing against stably expressed target genes.

## Materials and Methods

### Sample Collection

First to third parity, specific pathogen-free Yorkshire sows were used for this study (University of Guelph, Guelph, ON, Canada). The Animal Care Committee of the University of Guelph approved all procedures (Animal utilization protocol number 10RO61). Sows were checked daily for estrus using an intact boar. At estrus, sows were placed in stalls and bred by artificial insemination using fresh pooled semen. Sows were re-bred 24 hours later. At gestation day 20 (gd20), sows were euthanized (n = 3). Reproductive tracts were collected at the University of Guelph abattoir and transported to the laboratory on ice. The uteri were cut longitudinally along the anti-mesometrial side to expose conceptuses. One healthy, and one arresting conceptus were selected per sow, based on disparity in size and vascularity as previously described [24], [25], [33]. Paired samples of mesometrial endometrium and trophoblast were collected from each gd20 attachment site. Non-pregnant samples were collected from random, mesometrial endometrial sites from mid-estrus sows (n = 4). Samples were immediately frozen and stored at −80°C.

### miRNA Extraction

Samples were thawed on ice, and total RNA including miRNA was extracted from all samples using miRNeasy mini kit (Qiagen, Mississauga, ON, Canada) according to the manufacturer's directions. Briefly, 30 mg of frozen tissue was placed in 700 µl of QIAzol lysis reagent from the kit, and disrupted using a rotor-stator homogenizer and Kontes pestles (Fisher Scientific, Ottawa, ON, Canada) for 30 seconds at room temperature. The mixture was allowed to sit for 5 min, and 140 µl of chloroform (Fisher Scientific, Ottawa, ON, Canada) was added to the tube. Tubes were vigorously shaken by vortex for 15 seconds and allowed to settle for 3 minutes at room temperature. Samples were centrifuged at 4°C at 12000 g for 15 minutes. The aqueous phase of the mixture was added to 525 µl of 100% ethanol (University of Guelph, Guelph, ON, Canada) and pipetted several times to mix. The mixture was then transferred to an RNeasy mini spin column, and spun at 8000 g for 15 seconds, at room temperature. The flow-through was discarded. The columns were washed with 700 µl of Buffer RWT, and centrifuged at 8000 g for 15 seconds followed by wash in 500 µl of Buffer RPE. Flow-through was discarded. Finally, columns were transferred to new collecting tubes provided in the kit, and 30 µl of DNase/RNase free water (Gibco, Burlington, ON, Canada) was added directly on the column membrane and toral RNA was eluted by centrifugation at 8000 g for 1 minute. The concentration and purity of the RNA extracted was measured using the GeneQuant pro RNA/DNA calculator (Biochrom Ltd., Cambridge, UK). Total RNA was stored at −80°C until required.

### miRNA cDNA Preparation

Total RNA including miRNA from each sample was reverse transcribed using the miScript Reverse Transcription kit (Qiagen, Mississauga, ON, Canada) as per the manufacturer's protocol. Briefly, a master mix of 4 µl of miScript RT Buffer, and 1 µl of miScript Reverse Transcriptase Mix per tube was prepared and distributed to 0.2 mL PCR tubes (UltiDent Scientific, St. Laurent, QC, Canada) on ice. DNase/RNase free water was added to 1 µg of RNA to bring the volume to 15 µl. The RNA was added to the PCR tube, bringing the final volume to 20 µl. Samples were incubated at 37°C for 60 minutes and at 95°C for 5 minutes. The reaction mixture was placed on ice and diluted with 150 µl of DNase/RNase free water. The concentration and purity of the cDNA was measured using the GeneQuant pro RNA/DNA calculator. cDNA was stored at −80°C until required.

### Reference and Target Genes

All six reference genes (*RNU1A*, *RNU5A*, *RNU6B*, *SNORD25*, *SCARNA17*, and *SNORA73A*) were selected based on the availability of commercial primers. They are all short, non-coding RNAs of roughly 150 bp in length [33], and are marketed for use in human, mouse, rat, and dog tissues by Qiagen (Qiagen, Mississauga, ON, Canada). Gene short forms, full names, and estimated product sizes are listed in Table 1.

Primers for porcine-specific target genes *ssc-miR-331-5p* and *ssc-miR-339-3p* were custom designed using sequences available in the miRBase version 16 [34], and ordered from Qiagen (Qiagen, Mississauga, ON, Canada). These genes were selected because of their stability between healthy endometrium and healthy trophoblast, as determined in the same experimental tissue set by microarray [unpublished].

### Real Time PCR

Primers for all six candidate and two target genes (Qiagen, Mississauga, ON, Canada) were diluted according to the manufacturer's protocol. A pool of miRNA cDNA was created using all samples. An initial real time PCR to test the primers was performed on pooled miRNA cDNA in triplicate using the miScript SYBR Green PCR kit (Qiagen, Mississauga, ON, Canada) in a capillary-based LightCycler (Roche Diagnostics, Laval, QC, Canada). The PCR conditions were set according to manufacturer's protocols (activation: 95°C; 15 minutes, 45 cycles of denaturation: 94°C; 15 sec., annealing: 55°C; 30 sec., and extention 70°C; 30 sec., melting curve: 70–95°C at a rate of 0.1°C per second). Products for each gene were serially diluted ten-fold to create a standard curve.

Each of the six candidate genes was measured in duplicate in all sixteen samples (n = 3 gd20 Healthy Endometrium (HE), n = 3 gd20 Arresting Endometrium (AE), n = 3 gd20 Healthy Trophoblast (HT), n = 3 gd20 Arresting Trophoblast (AT), and n = 4 Non-Pregnant Endometrium (NP)) by 384 well, plate-based real-time PCR (LC480, Roche Diagnostics, Laval, QC, Canada). C_p_ values were measured by LightCycler 480 software (release 1.5.0 SP3, Roche Diagnostics, Laval, QC, Canada) and averaged by tissue type. Target genes *miR-331-5p* and *miR-339-3p* were also measured in duplicate in the same HE and HT samples (n = 3 gd20 HE, n = 3 gd20 HT). Standard curves for each gene and an RT-negative control were also included on the plate. The PCR conditions were identical to those used in the capillary-based system, except for the melting curve: 65–97°C at a rate of 2.5°C per second. After the generation of 10-fold dilution series standard curves, the PCR efficiency was calculated for each reference gene using the LightCycler 480 software (Roche Diagnostics, Laval, QC, Canada). Several dilutions were then excluded from the standard curve to optimize PCR efficiency. Melting curve analysis was performed to ensure amplification of only one product and to ensure products melted in the appropriate range for miRNAs (∼74–77°C, according to the manufacturer: Qiagen, Mississauga, ON, Canada). Finally, PCR products were run on a 0.5% agarose gel to estimate product size.

### Cloning and Sequencing

Fresh PCR product was used for cloning to confirm primer specificity. All PCR products were inserted into plasmid vectors using the topoisomerase-TA cloning kit (Invitrogen Life Technologies, Burlington, ON, Canada) as per manufacturer's instructions. Bacterial colonies were grown at 37°C on LB media containing ampicillin, then transferred to liquid LB. Plasmid DNA was purified using the Genelute Plasmid Mini-Prep Kit (Sigma, St. Louis, MO, USA). Plasmids were then sent for sequencing at the Laboratory Services Division of the University of Guelph. Each sequence underwent BLASTN analysis on the National Center for Biotechnology Information website. Sequences were submitted to NCBI GenBank (*RNU1A*: JN617883; *RNU5A*: JN617884; *RNU6B*: JN617885; *SNORD25*: JN646111; *SCARNA17*: JN617886; *ssc-miR-331-5p*: JN646112; and *ssc-miR-339-3p*: JN646113). If the gene product could not be positively identified, cloning was repeated two more times.

### Data Analysis

C_p_s (from LightCycler software) were imported into geNorm software (http://medgen.ugent.be/~jvdesomp/genorm/) [26] and the expression stability of each reference gene was evaluated. M values represent the combined variation within the experimental group and between reference genes. Genes with the lowest M value have the most stable expression. V values were calculated to determine the optimal number of reference genes to use for subsequent quantifications.

Average C_p_s were converted into relative quantities (RQ) for NormFinder (http://www.mdl.dk/publicationsnormfinder.htm) analysis following the methods in Latham, 2010 [9]. The NormFinder algorithm independently estimates the inter- and intra-group variance, and provides a stability value for each reference gene. Genes with the lowest stability value have the most stable expression [27]. The correlation between the geNorm M value and the NormFinder Stability Value was evaluated by the coefficient of determination (R^2^) (SigmaPlot 10.0, Systat Software Inc., Chicago, IL, USA).

To determine reference gene stability across the experimental panel of samples, data was analyzed by one-way ANOVA (SigmaPlot 10.0, Systat Software Inc., Chicago, IL, USA), and the average expression level (as a C_p_) and SEM for each reference gene were calculated and plotted. For all statistical tests, a p value of <0.05 was considered significant.

The relative quantification of each target gene against each reference gene was calculated using LC480 software (release 1.5.0 SP3, Roche Diagnostics, Laval, QC, Canada). The fold change for each target gene was calculated using the geometric mean of *RNU1A/SNORD25* as a normalizer and the ddCt method [9], [35], [36]. HE samples were arbitrarily set to a value of 1 + SEM (as a percentage of the variation among biological replicates), HT samples show the fold change above or below HE + SEM . T-tests were used to determine statistical significance between HE and HT samples.

## References

[1] Lai EC (2002). Micro RNAs are complementary to 3′ UTR sequence motifs that mediate negative post-transcriptional regulation.. Nat Genet.

[2] Engels BM, Hutvagner G (2006). Principles and effects of microRNA-mediated post-transcriptional gene regulation.. Oncogene.

[3] Chen CZ, Li L, Lodish HF, Bartel DP (2004). MicroRNAs modulate hematopoietic lineage differentiation.. Science.

[4] Croce CM, Calin GA (2005). miRNAs, cancer, and stem cell division.. Cell.

[5] Ambros V (2004). The functions of animal microRNAs.. Nature.

[6] Bartel DP (2004). MicroRNAs: Genomics, biogenesis, mechanism, and function.. Cell.

[7] Esquela-Kerscher A, Slack FJ (2006). Oncomirs - microRNAs with a role in cancer.. Nat Rev Cancer.

[8] Lu J, Getz G, Miska EA, Alvarez-Saavedra E, Lamb J (2005). MicroRNA expression profiles classify human cancers.. Nature.

[9] Latham GJ (2010). Normalization of microRNA quantitative RT-PCR data in reduced scale experimental designs.. Methods Mol Biol.

[10] Peltier HJ, Latham GJ (2008). Normalization of microRNA expression levels in quantitative RT-PCR assays: Identification of suitable reference RNA targets in normal and cancerous human solid tissues.. RNA.

[11] Chang KH, Mestdagh P, Vandesompele J, Kerin MJ, Miller N (2010). MicroRNA expression profiling to identify and validate reference genes for relative quantification in colorectal cancer.. BMC Cancer.

[12] Davoren PA, McNeill RE, Lowery AJ, Kerin MJ, Miller N (2008). Identification of suitable endogenous control genes for microRNA gene expression analysis in human breast cancer.. BMC Mol Biol.

[13] Kulcheski FR, Marcelino-Guimaraes FC, Nepomuceno AL, Abdelnoor RV, Margis R (2010). The use of microRNAs as reference genes for quantitative polymerase chain reaction in soybean.. Anal Biochem.

[14] Alevizos I, Alexander S, Turner RJ, Illei GG (2011). MicroRNA expression profiles as biomarkers of minor salivary gland inflammation and dysfunction in sjogren's syndrome.. Arthritis Rheum.

[15] Yaman Agaoglu F, Kovancilar M, Dizdar Y, Darendeliler E, Holdenrieder S (2011). Investigation of miR-21, miR-141, and miR-221 in blood circulation of patients with prostate cancer.. Tumour Biol.

[16] Mahn R, Heukamp LC, Rogenhofer S, von Ruecker A, Muller SC (2011). Circulating microRNAs (miRNA) in serum of patients with prostate cancer.. Urology.

[17] Ng EK, Chong WW, Jin H, Lam EK, Shin VY (2009). Differential expression of microRNAs in plasma of patients with colorectal cancer: A potential marker for colorectal cancer screening.. Gut.

[18] Gee HE, Buffa FM, Camps C, Ramachandran A, Leek R (2011). The small-nucleolar RNAs commonly used for microRNA normalisation correlate with tumour pathology and prognosis.. Br J Cancer.

[19] Dheda K, Huggett JF, Bustin SA, Johnson MA, Rook G (2004). Validation of housekeeping genes for normalizing RNA expression in real-time PCR.. BioTechniques.

[20] Barber RD, Harmer DW, Coleman RA, Clark BJ (2005). GAPDH as a housekeeping gene: Analysis of GAPDH mRNA expression in a panel of 72 human tissues.. Physiol Genomics.

[21] Nygard AB, Jorgensen CB, Cirera S, Fredholm M (2007). Selection of reference genes for gene expression studies in pig tissues using SYBR green qPCR.. BMC Mol Biol.

[22] Mestdagh P, Van Vlierberghe P, De Weer A, Muth D, Westermann F (2009). A novel and universal method for microRNA RT-qPCR data normalization.. Genome Biol.

[23] Pope WF, Zavy MT, Geisert RD (1994). Embryonic mortality in swine.. Embryonic Mortality in Domestic Species.

[24] Tayade C, Black GP, Fang Y, Croy BA (2006). Differential gene expression in endometrium, endometrial lymphocytes, and trophoblasts during successful and abortive embryo implantation.. J Immunol.

[25] Tayade C, Fang Y, Hilchie D, Croy BA (2007). Lymphocyte contributions to altered endometrial angiogenesis during early and midgestation fetal loss.. J Leukoc Biol.

[26] Vandesompele J, De Preter K, Pattyn F, Poppe B, Van Roy N (2002). Accurate normalization of real-time quantitative RT-PCR data by geometric averaging of multiple internal control genes.. Genome Biol.

[27] Andersen CL, Jensen JL, Orntoft TF (2004). Normalization of real-time quantitative reverse transcription-PCR data: A model-based variance estimation approach to identify genes suited for normalization, applied to bladder and colon cancer data sets.. Cancer Res.

[28] Kuijk EW, du Puy L, van Tol HT, Haagsman HP, Colenbrander B (2007). Validation of reference genes for quantitative RT-PCR studies in porcine oocytes and preimplantation embryos.. BMC Dev Biol.

[29] Choong ML, Yang HH, McNiece I (2007). MicroRNA expression profiling during human cord blood-derived CD34 cell erythropoiesis.. Exp Hematol.

[30] Corney DC, Flesken-Nikitin A, Godwin AK, Wang W, Nikitin AY (2007). MicroRNA-34b and MicroRNA-34c are targets of p53 and cooperate in control of cell proliferation and adhesion-independent growth.. Cancer Res.

[31] Zhu HT, Dong QZ, Wang G, Zhou HJ, Ren N (2011). Identification of suitable reference genes for qRT-PCR analysis of circulating microRNAs in hepatitis B virus-infected patients.. Mol Biotechnol.

[32] Wotschofsky Z, Meyer HA, Jung M, Fendler A, Wagner I (2011). Reference genes for the relative quantification of microRNAs in renal cell carcinomas and their metastases.. Anal Biochem.

[33] Wessels JM, Linton NF, van den Heuvel MJ, Cnossen SA, Edwards AK (2011). Expression of chemokine decoy receptors and their ligands at the porcine maternal-fetal interface.. Immunol Cell Biol.

[34] QIAGEN (2011). Highly specific PCR products for miScript PCR controls in human, mouse, and rat.. http://www.qiagen.com/products/mirnacontrols.aspx#Tabs=t1.

[35] Griffiths-Jones S (2004). The microRNA registry.. Nucleic Acids Res.

[36] Livak KJ, Schmittgen TD (2001). Analysis of relative gene expression data using real-time quantitative PCR and the 2(-delta delta C(T)) method.. Methods.

